# Response of maize root endophytes to carbon-nitrogen coupling in the irrigated area of the West Liaohe Plain, Inner Mongolia

**DOI:** 10.1186/s12870-026-09641-0

**Published:** 2026-08-03

**Authors:** Chengcheng Wu, Rula Sa, Naijia Liu, Xingbao Chen, Junqi Chu, Jicheng Tai

**Affiliations:** 1 Agricultural College, Inner Mongolia Minzu University, Tongliao, Inner Mongolia 028000 China; 2 College of Grassland Science, Inner Mongolia Minzu University, Tongliao, Inner Mongolia 028000 China

**Keywords:** Maize root endophytes, Stover return, Nitrogen fertilizer, Silking stage, Relative quantification, Absolute quantification

## Abstract

Stover return and nitrogen management are key agricultural practices influencing crop root-associated microbial communities. However, how their coupling affects the community structure, absolute abundance, and functional potential of maize root endophytes remains poorly understood. This study was based on a five-year stationary field experiment in the West Liaohe Plain irrigation area, a two-factor split-plot design was established with two stover return levels (no return; half return at 4500 kg/ha; full return at 9000 kg/ha) and two nitrogen application rates (conventional 450 kg/ha; reduced 300 kg/ha), resulting in six treatments. Root samples were collected at the silking stage (R1). High-throughput sequencing of the 16 S rRNA gene V4-V5 region and the ITS gene ITS2 region, combined with an internal standard-based absolute quantification method, was used to characterize root endophytic bacterial and fungal community structure, diversity, absolute abundance, and their correlations with environmental factors. Results showed that the dominant bacterial phylum was Pseudomonadota, and the dominant genus was *Pseudomonas*. Compared with NN, NR significantly increased the relative abundance of *Pseudomonas* but reduced bacterial diversity. Although the total absolute abundance of the fungal community in NR was high, its species composition was homogeneous with strong homogeneity. In HN, Pseudomonadota dominated the bacterial community, with enrichment of organic matter-degrading taxa, while the abundance of potential pathogens such as *Fusarium* increased in fungi. HR significantly enriched Bacteroidota and suppressed *Pseudomonas*, containing the highest number of unique bacterial ASVs; its fungal community exhibited the strongest heterogeneity, with increased abundance of beneficial saprotrophs such as *Mortierella*. The bacterial community structure in TN was conservative, still dominated by Pseudomonadota, with the lowest total fungal abundance but enrichment of yeast taxa. TR significantly weakened the dominance of *Pseudomonas* and increased the abundance of *Rhizobium*, *Agrobacterium*, and Actinomycetota, maintaining high functional differentiation potential, while fungi enriched saprotrophs including *Mortierella* and *Penicillium*. Correlation analysis indicated that alkali-hydrolyzable nitrogen and available potassium were the core environmental factors driving changes in root endophytic microbial community structure. In conclusion, full stover return combined with reduced nitrogen application (TR) facilitates the construction of a multifunctional community with both nitrogen-fixing potential and organic matter transformation capacity, providing a microbiological basis for the synergistic realization of maize stover resource utilization and nitrogen fertilizer reduction in the West Liaohe Plain irrigation area.

## Introduction

Sustainable development of agricultural ecosystems depends on the dynamic balance between resource use efficiency and environmental carrying capacity [1–2]. Stover return can increase soil organic matter content [3], improve aggregate structure [4], and enhance soil carbon sequestration potential [5]. When implemented in synergy with optimized nitrogen fertilization, it has become a core strategy for green production in China’s major grain-producing regions [6]. However, long-term stover removal or sole reliance on chemical fertilizers not only exacerbates soil degradation and microbial functional decline [7–8], while excessive nitrogen application intensifies environmental risks such as soil acidification [9–15] and water eutrophication [16], severely compromising the service functions of farmland ecosystems. Currently, nitrogen use efficiency in maize production in China remains at a low level of 30%–40% [17]. How to achieve nitrogen reduction without compromising yield is an urgent issue that must be addressed.

The synergistic input of carbon and nitrogen resources alters the nutrient distribution patterns and stoichiometric characteristics of the rhizosphere microenvironment [18], thereby driving the reassembly and functional evolution of soil microbial communities [19], and ultimately regulating the balance between nutrient mineralization and immobilization as well as crop productivity [20] Maize (*Zea mays* L.), the cereal crop with the largest sown area worldwide [21], has received extensive attention regarding the response of its rhizosphere microbiome to carbon and nitrogen management [22]. However, root endophytic microorganisms, which directly colonize internal root tissues and are more tightly coupled with host functions, remain insufficiently studied. In fact, endophytes perform ecological functions — such as promoting nutrient transport, suppressing soil-borne diseases, and buffering abiotic stresses — that rhizosphere free-living microorganisms cannot readily replace [23–25]. More critically, how the interactive effects between stover return gradient and nitrogen application level regulate the community assembly processes of root endophytic microorganisms remains poorly understood from a quantitative perspective [26–27]. This knowledge gap has become a theoretical bottleneck for precisely modulating the crop microbiome to achieve “carbon-driven nitrogen retention and yield enhancement with reduced nitrogen input.”

The absolute quantification technique developed in recent years, which simultaneously acquires relative and absolute abundance information of microorganisms, provides a means to more accurately reveal the true response patterns of microbial communities to environmental changes [28]. On this basis, the present study took a maize system in a typical irrigation area as the research object. A two-factor long-term field experiment was established with stover return amount and nitrogen application rate as factors. Using high-throughput amplicon sequencing combined with an absolute quantification method, we systematically examined the effects of carbon and nitrogen management on the community structure, diversity, and functional potential of maize root endophytic bacteria and fungi. We hypothesized that straw return combined with different nitrogen supply levels would drive niche differentiation of maize root endophytes. Specifically, nitrogen reduction would enhance host-mediated selective recruitment of specific microorganisms, leading to a significant decrease in bacterial alpha diversity and a simplification of interspecific interaction networks. Half stover return, constrained by limited carbon supply, would drive the niche expansion of facultative pathogens. Full stover return, by providing a rich supply of lignocellulose, would steer the fungal community toward a directed reassembly dominated by saprotrophic beneficial taxa. The root endophytic community of maize under full stover return coupled with reduced nitrogen application (TR) would exhibit significantly higher functional redundancy than the monopolistic dominant-type community (NR), thereby providing a more robust microecological buffer for reproductive growth at the silking stage (R1) and maintaining higher system functional stability under nitrogen-reduced conditions.

## Results and analysis

### Relative abundance and distribution of maize root endophytic bacteria

Based on relative quantification of bacterial communities (Fig. 1a), there were 268 shared ASVs across treatments. The number of unique ASVs per treatment was: HR (1382) > NN (1067) > TR (835) > HN (501) > TN (444) > NR (399). Under conventional nitrogen, the no-return treatment (NN) had the most unique ASVs (1067). Under reduced nitrogen, the half-return treatment (HR) had the highest number of unique ASVs (1382), while the no-return treatment (NR) had the lowest (399), indicating a simplified community.At the phylum level (Fig. 1b), Pseudomonadota was the dominant phylum (59.45%-80.1%). Its abundance was higher in NR (80.10%), HN (79.26%), and TN (79.11%) than in HR (59.45%). Bacillota (5.34%-19.63%) and Bacteroidota (6.49%-11.97%) were secondary dominant phyla. Bacteroidota was enriched in HR (11.97%) and TN (9.45%). Bacillota was highest in NN (19.63%). Actinomycetota remained at high abundance in TR (8.35%) and HR (8.22%). HR specifically showed relatively high levels of Chloroflexota (2.25%), Planctomycetota (1.63%), and Acidobacteriota (1.77%).Under conventional nitrogen, NN had lower Pseudomonadota (61.90%) but higher Bacillota (19.63%). Both HN (79.26%) and TN (79.11%) had high Pseudomonadota, but TN had lower Actinomycetota (2.53%) than HN (4.80%). Under reduced nitrogen, full return slightly reduced Pseudomonadota, while no return increased it. Return treatments enhanced Bacillota competition and raised Actinomycetota abundance. HR increased Bacteroidota and rare groups, with higher relative abundance of Chloroflexota, though its ecological function needs verification.At the genus level (Fig. 1c), *Pseudomonas* was the dominant genus (15.19%-50.62%). It was higher in NR (50.62%), HN (38.47%), and TR (31.42%) but lower in HR (15.19%). *Priestia* was higher in TR (10.71%) and NN (10.45%). *Streptomyces* was enriched in HR (5.25%), TR (4.50%), and NR (4.26%). Under conventional nitrogen, NN had lower *Pseudomonas* (19.32%) but higher *Priestia* (10.45%) and *Rhizobium* (2.06%). HN was dominated by *Pseudomonas* (38.47%). Under reduced nitrogen, NR was dominated by *Pseudomonas* (50.62%) with a simplified community. HR suppressed *Pseudomonas* (15.19%) and increased rare groups like *Streptomyces* (5.25%). TR maintained high *Pseudomonas* (31.42%) and also enriched *Streptomyces* (4.50%) and *Rhizobium* (2.82%). Return treatments promoted *Streptomyces* and rhizobia-related genera. In HR, genera like *Flavobacterium* (1.04%) and *Chitinophagaceae_genus* (2.29%) also increased.Diversity indices (Fig. 1d) showed that the Observed richness ranked as: HR (1579) > NN (1274) > TR (1253) > HN (881) > NR (833) > TN (658). HR was significantly higher than other treatments (*P* < 0.05). Shannon index followed the same order (*P* < 0.001). Simpson index was opposite, with TN highest (0.177, *P* < 0.05). Coverage index showed no significant differences (*P* > 0.05). PCoA based on Weighted UniFrac distance (Fig. 1e) showed that PC1 and PC2 explained 42.03% and 14.36% of variation. HN and HR clustered on the positive side of PC1, with similar structures. NN, NR, and TN were on the negative side of PC1, with TN shifting on PC2. TR partly clustered with half-return treatments, suggesting shared environmental influences. PERMANOVA analysis (Fig. 1f) confirmed that bacterial community structures differed among treatments (*P* < 0.001).

**Fig. 1:**
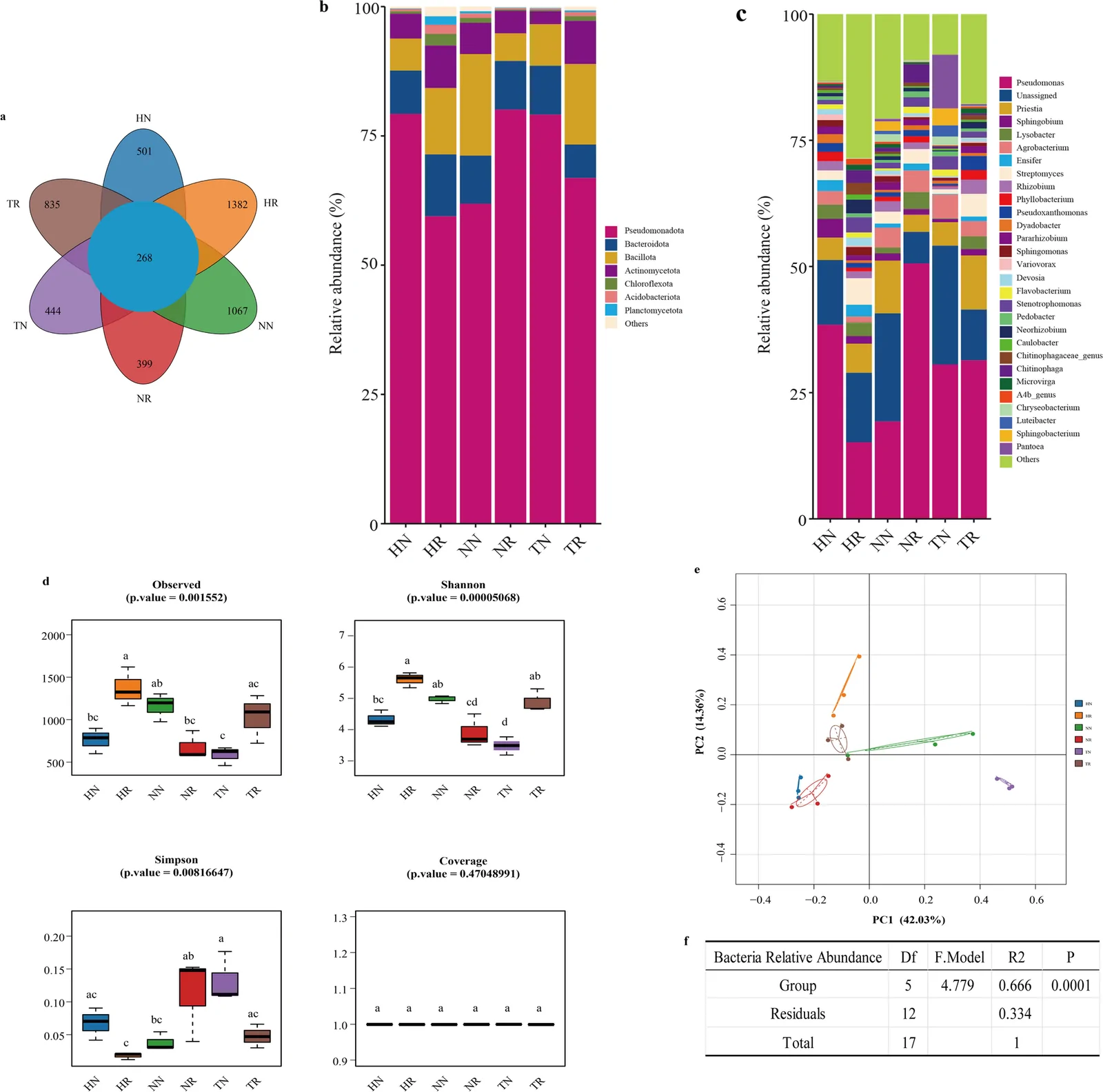
Community structure of maize root endophytic bacteria under different nitrogen application and stover return treatments.(relative quantification). Note: **a **ASV Venn diagram of bacterial communities based on relative quantification, each color represents a treatment group. Overlapping areas indicate shared ASVs, while non-overlapping areas represent unique ASVs of each treatment group.; **b **Relative abundance at the phylum level; **c **Relative abundance at the genus level; **d **Alpha diversity indices. In the boxplots, the box represents the interquartile range (IQR), the horizontal line inside the box indicates the median, and the whiskers extend to Q3 + 1.5 × IQR and Q1–1.5 × IQR, respectively. Points beyond the whiskers are outliers; **e **PCoA analysis based on Weighted UniFrac distance (relative quantification), each point represents a sample, colors correspond to different treatment groups, and the distance between points reflects community similarity; **f **PERMANOVA analysis of the relative abundance of bacteria. Note: In figure (**a**), each color represents a treatment group, overlapping areas indicate the number of shared ASVs, and non-overlapping portions represent the number of unique ASVs in each treatment group. In figure (**e**), each point represents an individual sample, colors correspond to different treatment groups, and the distance between points reflects community similarity

### Relative abundance and distribution of maize root endophytic fungi

Based on ASV-level Venn analysis (Fig. 2a), there were 74 shared ASVs across treatments. The number of unique ASVs per treatment was: HR (312) > TR (240) > NN (188) > TN (172) > HN (157) > NR (152). This shows that reduced nitrogen combined with stover return increased fungal community heterogeneity.At the phylum level (Fig. 2b), fungal communities were mainly composed of Ascomycota (23.79%-66.51%), Mucoromycota (9.17%-30.33%), Basidiomycota (2.10%-26.20%), and Mortierellomycota (1.03%-5.08%). Ascomycota was the dominant phylum across all treatments. Its relative abundance varied: it was high in TR (66.51%), HR (62.19%), HN (56.57%), and NR (54.50%), but low in TN (23.79%). Mucoromycota was enriched in TN (30.33%) and NR (28.89%). Mortierellomycota was high in TR (5.08%) and HR (3.65%). Basidiomycota was higher in TN (26.20%) and NN (24.83%). Low-abundance phyla like Chytridiomycota, Glomeromycota, and Rozellomycota were also detected in HR and TR.At the genus level (Fig. 2c), the community consisted of a few dominant genera and many low-abundance ones. Dominant genera included *Fusarium* (3.74%-36.94%), *Actinomucor* (6.06%-29.14%), *Tausonia* (1.46%-17.05%), *Mucor* (0.15%-12.11%), Rhizopus (0.15%-4.31%), and *Mortierella* (0.63%-4.54%). Under conventional nitrogen, NN was dominated by Actinomucor (15.76%) and Mucor (12.11%), with a relatively concentrated structure. HN had high relative abundance of *Fusarium* (36.92%). Under reduced nitrogen, NR had highly concentrated *Fusarium* (36.94%) and Actinomucor (23.60%). In HR, the proportions of *Curvularia* (10.31%), *Exophiala* (9.01%), and *Podospora* (3.18%) increased, along with more non-dominant and rare groups. TR maintained a high proportion of *Fusarium* (21.7%) but also enriched *Mortierella* (4.54%) and *Neocosmospora* (15.32%).Alpha diversity analysis (Fig. 2d) showed no significant differences in Observed, Shannon, Simpson, or Coverage indices among treatments (*P* > 0.05). PCoA analysis showed clear separation among treatments (Fig. 2e). PC1 and PC2 explained 26.23% and 15.31% of variation. HR and TR were clearly separated from other treatments, with greater within-group dispersion. HN and NN were relatively close. NR and TN were more concentrated, with less variation. Both also had lower diversity indices. PERMANOVA analysis (Fig. 2f) confirmed that fungal community structures differed among treatments (*P* < 0.001).

**Fig. 2:**
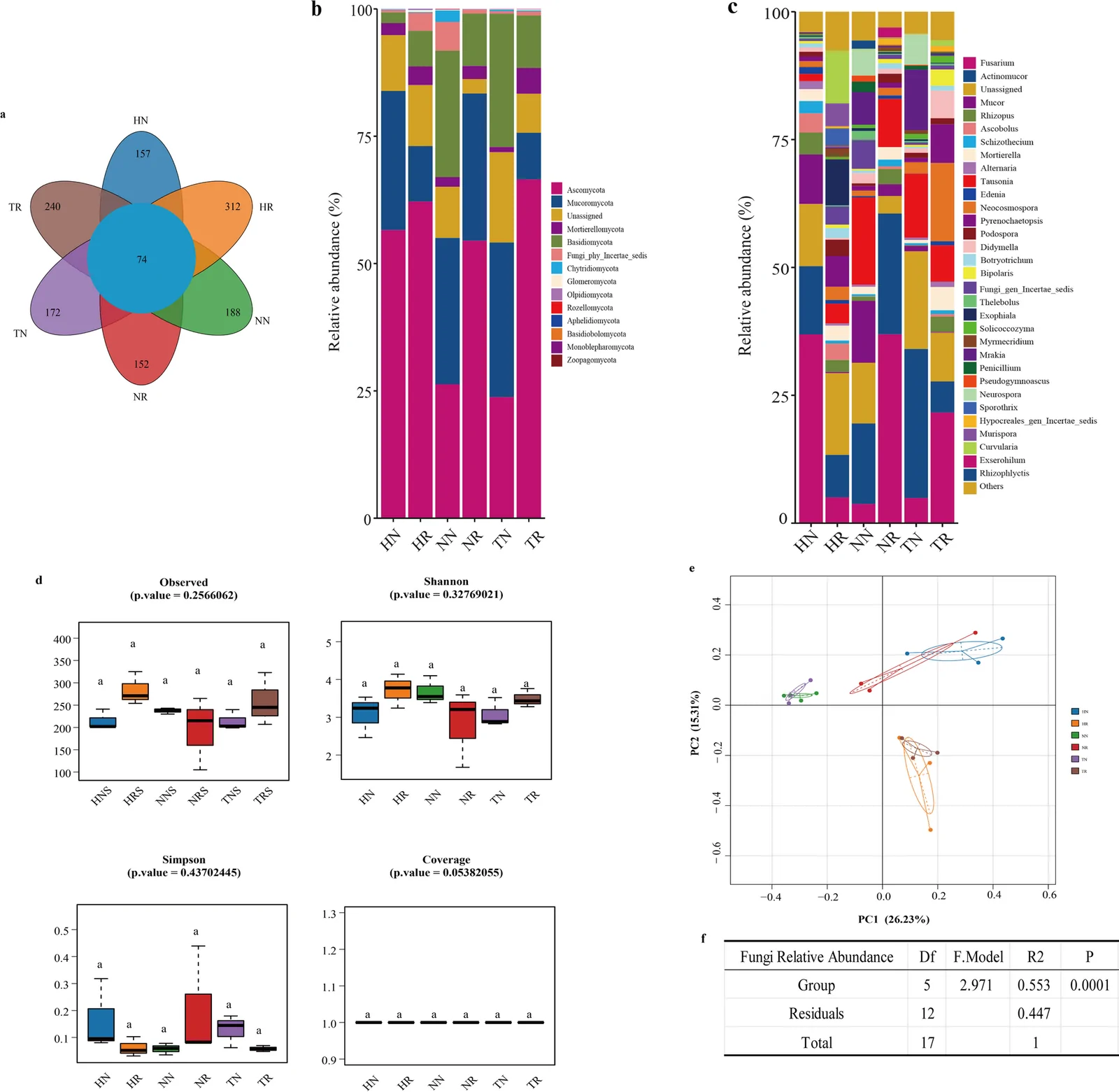
Community structure of maize root endophytic fungi under different nitrogen application and stover return treatments (relative quantification). Note: **a **ASV Venn diagram of fungal communities based on relative quantification; **b** Relative abundance of fungal communities at the phylum level; **c** Relative abundance of fungal communities at the genus level; **d** Alpha diversity indices. In the boxplots, the box represents the interquartile range (IQR), the horizontal line inside the box indicates the median, and the whiskers extend to Q3 + 1.5 × IQR and Q1–1.5 × IQR, respectively. Points beyond the whiskers are outliers; **e** PCoA based on weighted UniFrac distance (relative abundance). **f** PERMANOVA analysis of relative abundance of fungi

### Absolute abundance and distribution of maize root endophytic bacteria

Absolute quantification results (Fig. 3a) were highly consistent with relative quantification in terms of shared ASVs (272), ranking of unique ASVs, and diversity indices: HR (1413) > NN (1100) > TR (848) > HN (517) > TN (462) > NR (432). However, absolute quantification further revealed that TR maintained a high total ASV count (848) despite having a moderate number of unique ASVs. It also showed a balanced coexistence of Actinomycetota (5.12 × 10⁶–1.17 × 10⁷ copies/g) and Bacteroidota (8.40 × 10⁶–2.85 × 10⁷ copies/g).At the phylum level (Fig. 3b), Pseudomonadota remained the dominant phylum (7.64 × 10⁷–2.11 × 10⁸ copies/g). The peak of Bacillota in NN was significant (9.92 × 10⁶ copies/g). The enrichment of Acidobacteriota (1.03 × 10⁷ copies/g) and Chloroflexota (8.01 × 10⁶ copies/g) in HR was also more clearly shown.At the genus level (Fig. 3c), absolute quantification identified 37 taxa. NN had relatively high levels of *Priestia* (5.78 × 10⁶ copies/g) and *Agrobacterium* (2.34 × 10⁷ copies/g). Under reduced nitrogen, NR showed strong dominance of *Pseudomonas* (9.49 × 10⁷ copies/g), indicating community simplification. The increase of rare groups like *Devosia* (4.59 × 10⁶ copies/g) in HR was also quantitatively clearer.Alpha diversity analysis (Fig. 3d) showed that Observed richness ranked as: HR (1618) > NN (1297) > TR (1273) > HN (892) > NR (871) > TN (664). HR was significantly higher than others (*P* < 0.05). Shannon index ranked as: HR (5.92) > TR (5.43) > NN (5.35) > HN (4.85) > NR (4.52) > TN (4.23). HR was significantly higher than others (*P* < 0.001). Simpson index ranked as: NR (0.12) > TN (0.11) > HN (0.04) > TR (0.03) > NN (0.02) > HR (0.01). TN was significantly higher than others (*P* < 0.05). Coverage index showed differences among treatments (*P* < 0.001). PCoA analysis (Fig. 3e) showed a similar overall distribution to relative quantification, but PC1 and PC2 explained 34.18% and 15.09% of variation. HN and HR were farther apart on PC2, and TR was more independent. PERMANOVA analysis (Fig. 3f) confirmed that bacterial community structures differed among treatments (*P* < 0.001).

**Fig. 3:**
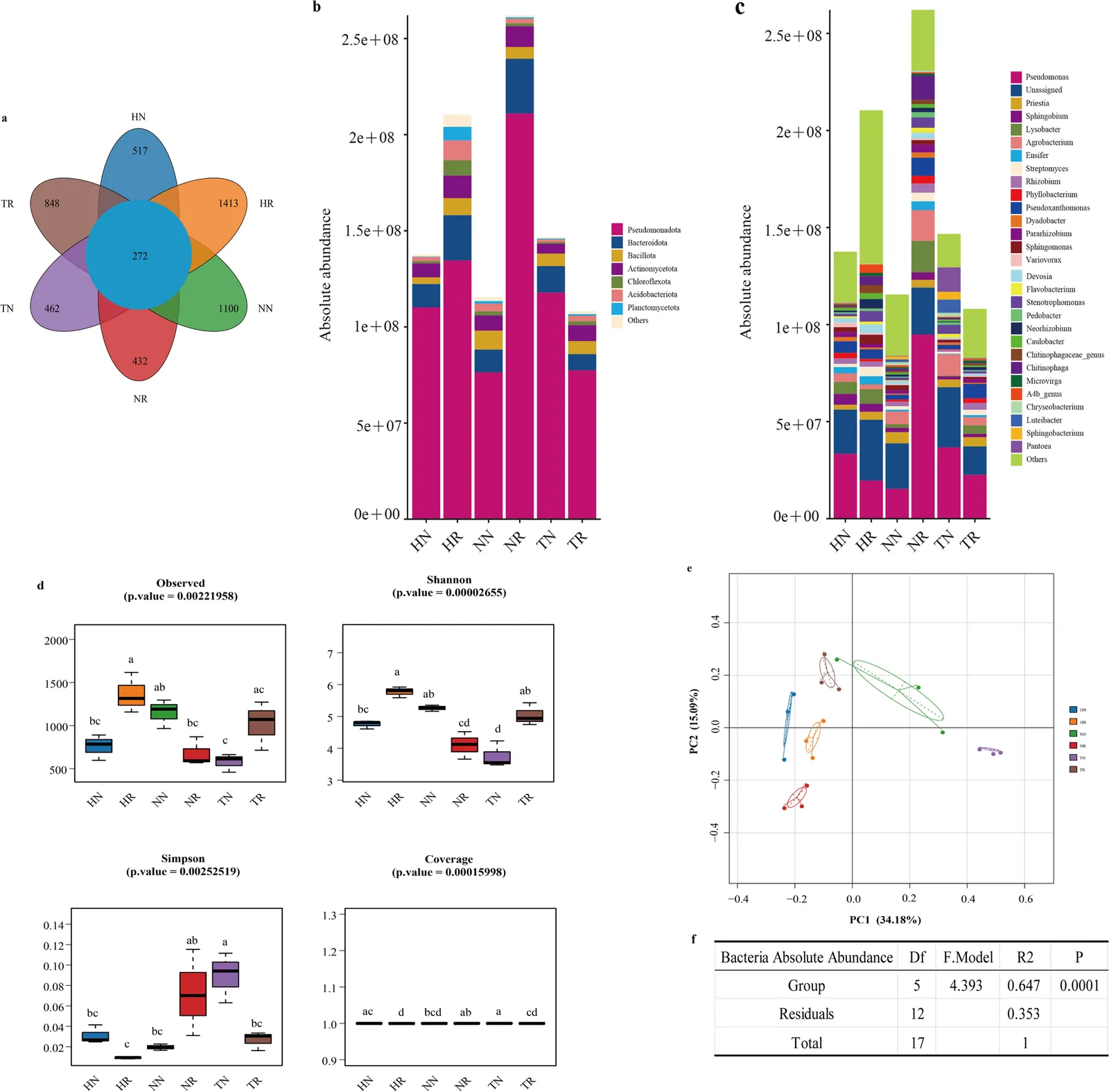
Community structure of maize root endophytic bacteria under different nitrogen application and stover return treatments (absolute quantification). Note: **a** ASV Venn diagram of bacterial communities based on absolute quantification; **b** Absolute abundance at the phylum level; **c** Absolute abundance at the genus level; **d** Alpha diversity indices. In the boxplots, the box represents the interquartile range (IQR), the horizontal line inside the box indicates the median, and the whiskers extend to Q3 + 1.5 × IQR and Q1–1.5 × IQR, respectively. Points beyond the whiskers are outliers; **e** PCoA analysis based on Weighted UniFrac distance (absolute quantification); **f** PERMANOVA analysis of the absolute abundance of bacteria. Figure annotations are the same as in Fig. 1

### Absolute abundance and distribution of maize root endophytic fungi

ASV-level Venn analysis (Fig. 4a) showed 74 shared ASVs across treatments, consistent with relative quantification. The ranking of unique ASVs was: HR (294) > TR (223) > TN (171) > NN (166) > NR (151) > HN (138).At the phylum level (Fig. 4b), Ascomycota was the main driver of community abundance (1.53 × 10⁶–1.48 × 10⁷ copies/g). NR had the highest total absolute abundance (1.48 × 10⁷ copies/g), mainly due to increases in Ascomycota and Mucoromycota (3.98 × 10⁶ copies/g). TN had the lowest total abundance, with Ascomycota (1.53 × 10⁶ copies/g) clearly reduced. Mortierellomycota remained high in TR (3.48 × 10⁵ copies/g). Glomeromycota was higher in HR (2.72 × 10⁴ copies/g), and Mucoromycota was higher in NR (3.98 × 10⁶ copies/g).At the genus level (Fig. 4c), NR had the highest total absolute abundance, with enriched groups like *Fusarium* (2.87 × 10⁵–1.22 × 10⁷ copies/g) and *Actinomucor* (3.83 × 10⁵–2.94 × 10⁶ copies/g). Under conventional nitrogen, NN and HN had relatively balanced distributions of dominant genera. In TN, most genera had low absolute abundance. HR and TR increased total abundance while also raising the absolute numbers of fungi like *Mortierella*, *Podospora*, *Didymella*, and *Penicillium*.Alpha diversity analysis (Fig. 4d) showed no significant differences among treatments in Observed, Shannon, Simpson, or Coverage indices (*P* > 0.05). PCoA based on Bray-Curtis distance (Fig. 4e) showed separation among treatments. PC1 and PC2 explained 20.47% and 13.43% of variation. Unlike relative quantification, NR was separated along PC1, while conventional nitrogen groups were more concentrated. PERMANOVA analysis (Fig. 4f) confirmed that fungal community structures differed among treatments (*P* < 0.001).

**Fig. 4:**
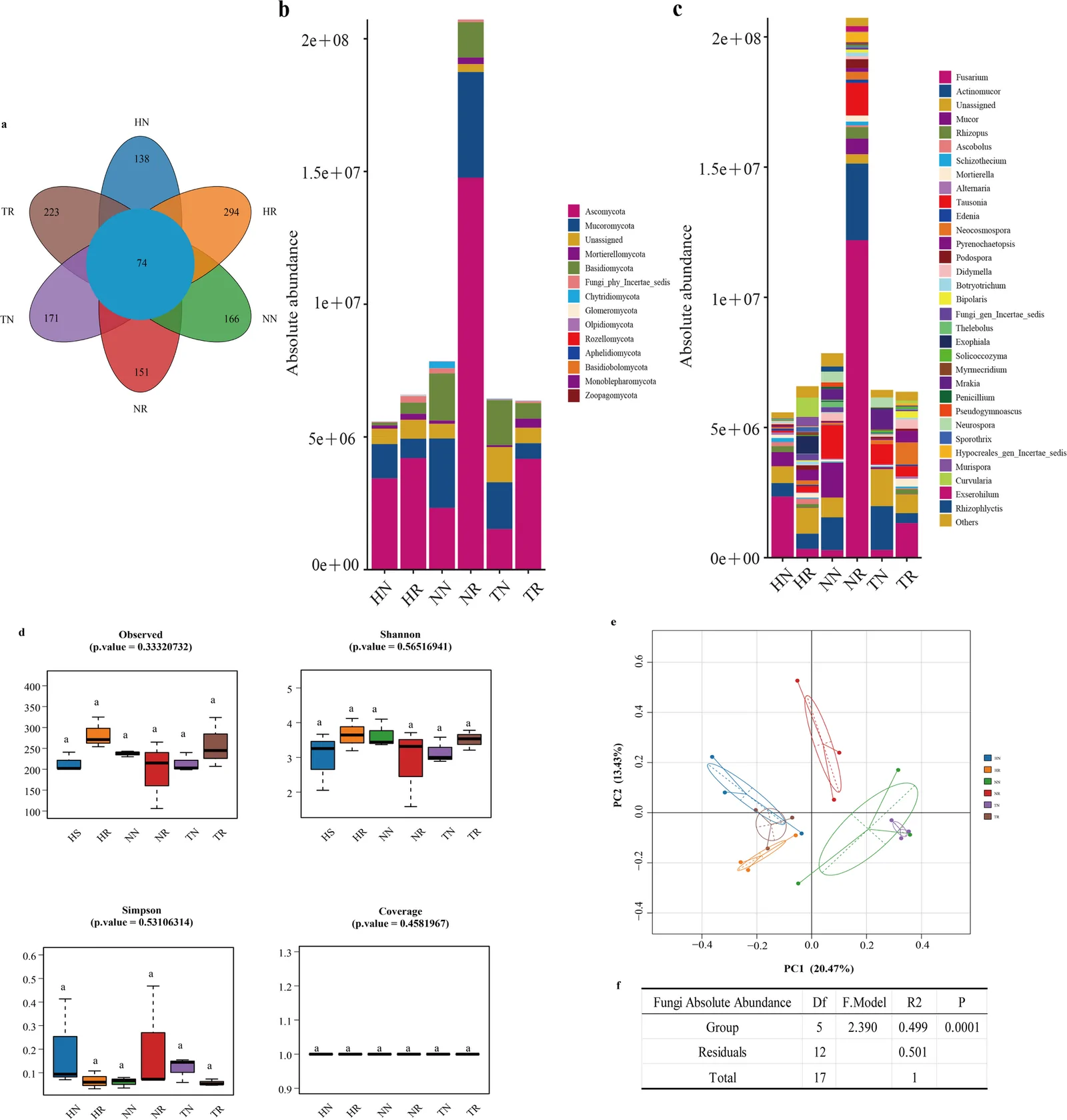
Community structure of maize root endophytic fungi under different nitrogen application and stover return treatments (absolute quantification). Note: **a** ASV Venn diagram of fungal communities based on absolute quantification; **b** Absolute abundance at the phylum level; **c** Absolute abundance at the genus level; **d** Alpha diversity indices (based on absolute quantification). In the boxplots, the box represents the interquartile range (IQR), the horizontal line inside the box indicates the median, and the whiskers extend to Q3 + 1.5 × IQR and Q1–1.5 × IQR, respectively. Points beyond the whiskers are outliers; **e** PCoA analysis based on Bray-Curtis distance (absolute quantification); **f** PERMANOVA analysis of the absolute abundance of fungi. Figure annotations are the same as in Fig. 1

### Differential analysis of root endophytes

LEfSe analysis (Fig. 5a) showed indicator taxa with significant differences in root endophytic bacterial communities among treatments. NN significantly enriched *Desulfuromonadaceae*, *Blastocatellaceae*, *Alphaproteobacteria*, and *Eremiobacteria*. HN mainly enriched *Natronincolaceae*, *Phyllobacteriaceae*, and *BD72BR169*. TR significantly enriched *Terriglobales* and *Candidatus*_*Koribacter*. HR mainly enriched *Holophaga* and *Planctomicrobium*. TN specifically recruited *Azospira*_*restricta*, *Gemmatimonas*, *Alcaligenaceae*, and *Azospira*. NR mainly enriched *KF_JG30_C25* and *Lysobacter*.LEfSe analysis for fungi (Fig. 5b) showed that TR mainly enriched *Basidiomycota*, *Cystofilobasidiales*, *Tremellomycetes*, *Mrakiaceae*, and *Tausonia_pullulans*. TN significantly enriched *Volvariella*, *Pluteaceae*, and *Sebacinales*. NR mainly enriched *Pseudoechria_decidua*, *Pseudoechria*, *Conocybe_papillata*, and *Glomeromycota*. NN significantly enriched *Exserohilum*, *Claroideoglomus_claroideum*, and *Sodiomyces*. HR mainly enriched *Cantharellales*, Minimedusa, and *Hydnaceae*. HN specifically enriched *Rhizophlyctis_rosea*, Apiosordaria, *Apiosordaria_effusa*, and *Knufia*.

The bacterial cladogram (Fig. 5c) showed differential nodes including *Candidatus_Koribacter*, *Koribacteraceae*, *Terriglobales*, *Blastocatellaceae*, *Natroninocalceae*, *Cytophagaceae*, *Nibribacter*, *Sphingobacteriaceae*, *Leptolyngbya_EcFYyyy_00*, *Berkelbacteria*, *Candidatus_Curtissbacteria*, *Elsterales*, *Devosia*, *Devosiaceae*, *Alcaligenaceae*, *Limnobacter*, *Methylotenera*, *Azospira*, *Enterobacteriales*, *Lysobacter*, *Permianibacter*, *Desulfuromonadales*, and environmental *clades AB1*, *BD72BR169*, *HOC36*, and *KF_JG30_C25*. The fungal cladogram (Fig. 5d) showed differential nodes including *Exserohilum*, *Sagenomella*, *Trichocomaceae*, *Hyalobilia*, *Pezizomycotina*, *Coniochaetaceae*, *Coniochaetales*, *Brunneochlamydosporium*, *Sodimonyces*, *Mymrecidium*, *Mymrecridiaceae*, *Mymrecridiales*, *Trichocladium*, *Apiosordaria*, *Pseudoechria*, *Sordariales*, *Volvariella*, *Pluteaceae*, *Minimedusa*, *Hydnaceae*, *Cantharellales*, *Waitea*, *Corticiaceae*, *Corticiales*, *Sebacinales*, *Agaricomycetes*, *Mrakia*, *Tausonia*, *Mrakiaceae*, *Cystofilobasidiales*, *Tremellomycetes*, *Blastocladiomycetes*, *Diversispora*, *Diversisporaceae*, *Diversisporales*, *Glomeromycetes*, *GS05*, and *Rozellomycotina*.

**Fig. 5:**
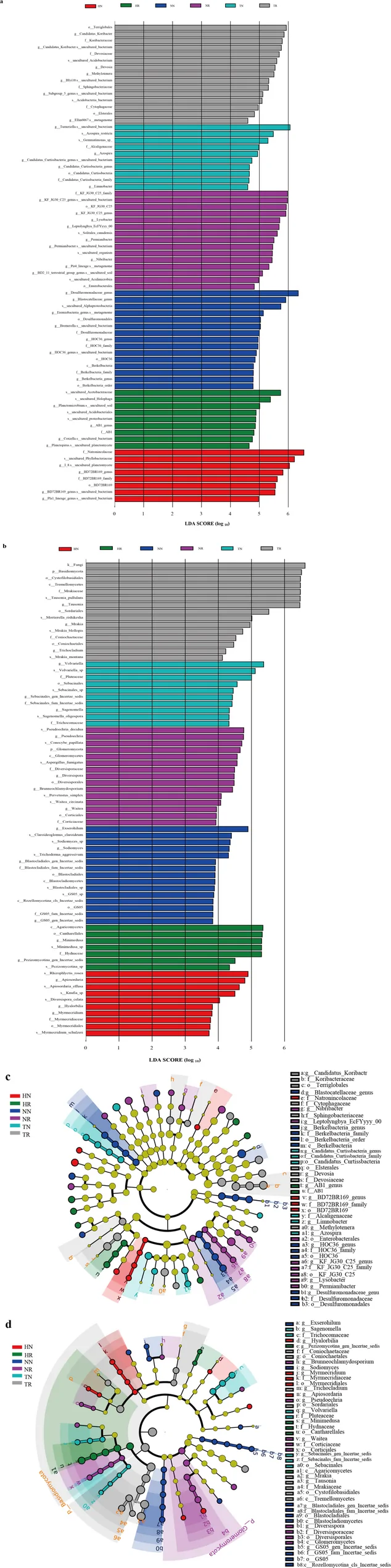
LEfSe analysis of maize root endophytic bacteria (**a**) and fungi (**b**) based on absolute quantification and corresponding cladograms for bacteria (**c**) and fungi (**d**). Note: Different colors represent different treatments. Red nodes in the cladogram indicate species with significantly greater abundance in the red group, green nodes indicate species with significantly greater abundance in the green group, and yellow nodes indicate species with no significant differences in abundance between groups. The node diameter is proportional to the abundance. Nodes from the inner to outer layers represent phylum, class, order, family, genus, and species. Annotations for each layer from outer to inner represent phylum, class, order, family, genus, and species; phylum-level annotations are shown on the outermost ring, whereas annotations for other levels are shown in the legend on the right. Only differential taxa with relative abundance > 0.1% and LDA score > 2.0 are shown in the figure. The remaining taxa are included in “Others” and are not displayed

### Functional potential analysis of root endophytes

For bacterial functions, chemoheterotrophy and aerobic chemoheterotrophy were the core functional groups. Their relative and absolute abundances were highest in NR (ANOVA, *P* < 0.05, Fig. 6a). Kruskal-Wallis tests further showed that nitrate reduction was significantly enriched in NR and HR (*P* < 0.05), while hydrocarbon degradation was more active in NN (*P* < 0.05, Fig. 6b).For fungal functions, Soil Saprotroph–Undefined Saprotroph was the dominant functional group. Its relative and absolute abundances were significantly higher in TN than in other treatments (ANOVA, *P* < 0.01, Fig. 6c). Kruskal-Wallis tests further showed that Plant Pathogen–Wood Saprotroph was significantly enriched in TR, with higher absolute abundance (*P* < 0.05). Pathogen-related groups like Animal Pathogen-Plant Pathogen also differed significantly among treatments (*P* < 0.05, Fig. 6d).

**Fig. 6:**
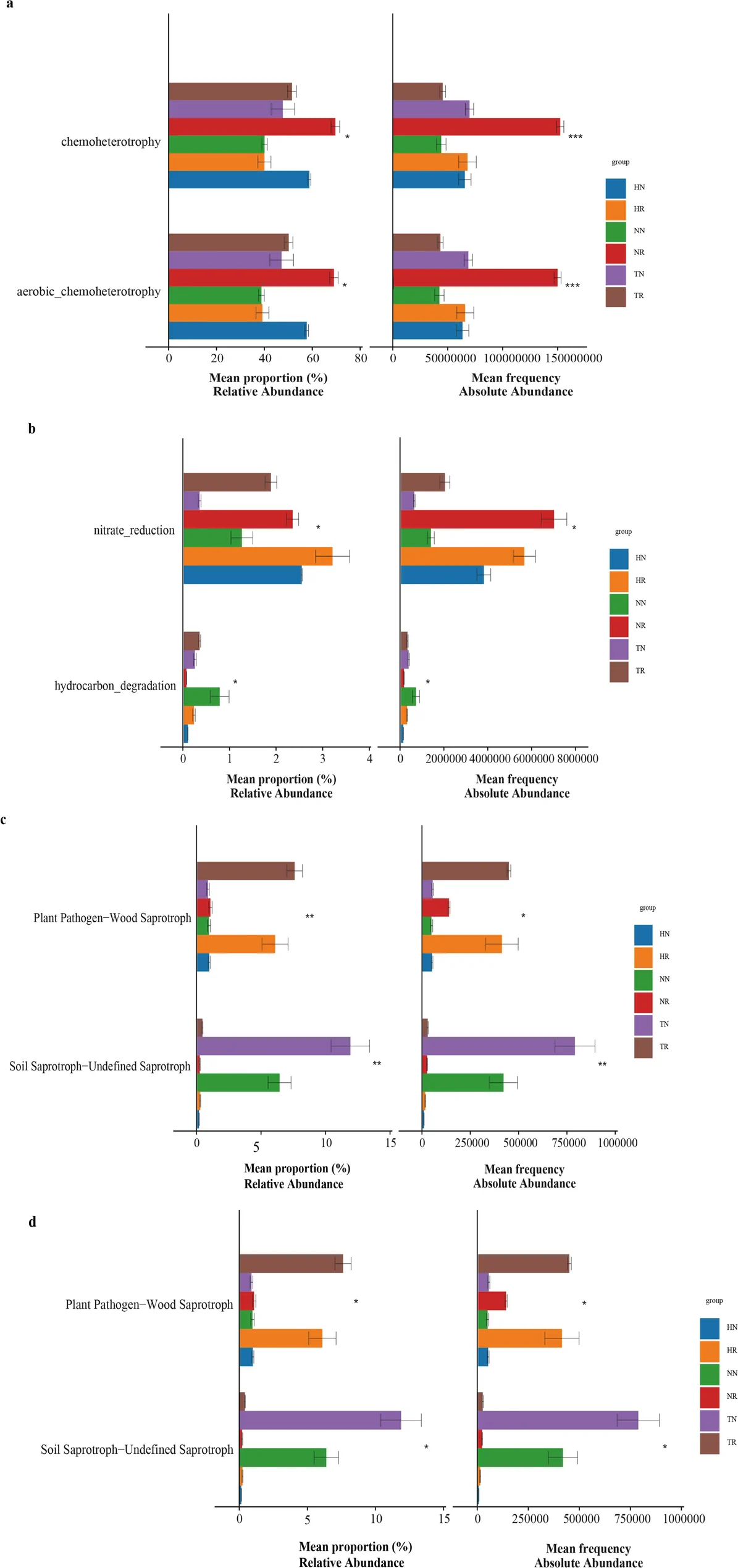
ANOVA and Kruskal–Wallis analysis of bacterial and fungal functional groups under different treatments. Note: **a** Relative abundance (mean proportion) and absolute abundance (mean frequency) of major bacterial functional groups; **b** Absolute abundance of bacterial functional groups involved in carbon and nitrogen metabolism; **c** Relative abundance and absolute abundance of major fungal functional groups; **d** Absolute abundance of fungal pathogen- and saprotroph-related functional groups. Significance marks displayed above the bars indicate overall differences among all treatment groups. Error bars represent mean ± standard error (SE). “*” and “**” indicate significant correlations at *P* < 0.05 and *P* < 0.01, respectively

### Relationship between root endophytic bacterial communities and environmental factors

Mantel test results (Fig. 7a) showed that available potassium (AK) and alkali-hydrolyzable nitrogen (AN) were the core environmental factors driving root endophytic microbial community assembly. Relative and absolute diversity of bacteria were significantly correlated with AN (*P* < 0.01, 0.25 ≤ *r* < 0.5). Absolute composition of bacteria was also significantly correlated with AN (*P* < 0.05, 0.25 ≤ *r* < 0.5). Relative and absolute diversity of fungi were mainly influenced by AK (*P* < 0.05, *r* ≥ 0.5). The correlations of fungal relative and absolute composition with AK were highly significant (*P* < 0.01, 0.25 ≤ *r* < 0.5). Soil pH, AP, SOC, and TN showed no significant direct effects on root endophytic diversity or composition (*P* > 0.05).

As shown in Fig. 7b, several bacterial genera were significantly correlated with soil nutrients (AP, TN, AK, AN). AP was significantly positively correlated with *Chryseobacterium*, *Oligoflexus*, *Legionella*, *Terribacillus*, *Mucilaginibacter*, *Mycobacterium*, *Curtobacterium*, *Rhodopseudomonas*, *Oligoflexaceae*, *Novosphingobium*, and *Sphingopyxis*. It was negatively correlated with *Promicromonospora*, *Rhizobacter*, *Glycomyces*, and *Phyllobacterium*. TN was positively correlated with *Fluviicola*, and positively correlated with *Nitrosomonas*, *AB1_genus*,* Taibaiella*, *Escherichia−Shigella*, *UCG − 005*, *Luteolibacter*, and *Rhodothermaceae*. AK was positively correlated with *Candidatus_Alysiosphaera*, *Promicromonospora*, *PAUC26f*, and *Terrabacter*, and negatively correlated with *Desertimonas* and *Stenotrophomonas*. AN was positively correlated with *Cystobacter* and PAUC26f, positively correlated with *Luteibacter*, *Xanthomonas*, *Paraburkholderia*, and *Patulibacte*r, and negatively correlated with *Adhaeribacter*, *Ardenticatenales*, *KD4−96_genus*, *Longimicrobiaceae*, *Polaromonas*, *Saccharimonadales*, *Aridibacter*, *Metabacillus*, *Ilumatobacteraceae*, *Flavitalea*, *Anaerolineaceae*, *Hyphomicrobium*, *Rhizorhabdus*, *Litchfieldia*, and *Rokubacteriales*.

As shown in Fig. 7c, several fungal genera were significantly correlated with soil nutrients (AP, TN, AK, AN). AP was positively correlated with *Hannaella*, and negatively correlated with *Lecythophora*, *Thanatephorus*, *Funneliformis*, *Dominikia*, *Rhizoctonia*, *Agrocybe*, *Lobulomycetales*, and *Striaticonidium*. TN was positively correlated with *Kurtzmanomyces*, and negatively correlated with *Fusicolla*, *Pseudogymnoascus*, *Solicoccozyma*, *Occultifur*, *Cladosporium*, *Paraphoma*, *Aspergillus*, *Pyrenochaeta*, *Cephalotrichum*, and *Hypocreales*. AK was positively correlated with *GS05*, *Leptosphaeria*, *Protocreopsis*, *Exophiala*, *Vishniacozyma*, *Rhizophlyctis*, *Neurospora*, *Naganishia*, *Picipes*, *Pseudomicrodochium*, *Ustilago*, *Myrmecridium*, *Talaromyces*,* Mrakia*, *Saitozyma*, and *Nectriopsis*, and negatively correlated with *Rhizopus*,* Fusarium*, *Lecythophora*, *Malassezia*, *Murispora*, and *Albifimbria*. AN was positively correlated with *Cephalotrichum*, *Filobasidium*, *Dictyosporiaceae*, *Eucasphaeria*, *Conocybe*, *Trichoderma*, *Meyerozyma*, *Oliveonia*, *Cladosporium*, *Niesslia*, *Lobulomycetales*, and *Striaticonidium*, and negatively correlated with *Trichocladium*, *Hannaella*, *Plectosphaerella*, and *Pseudocyclothyriella*.

**Fig. 7:**
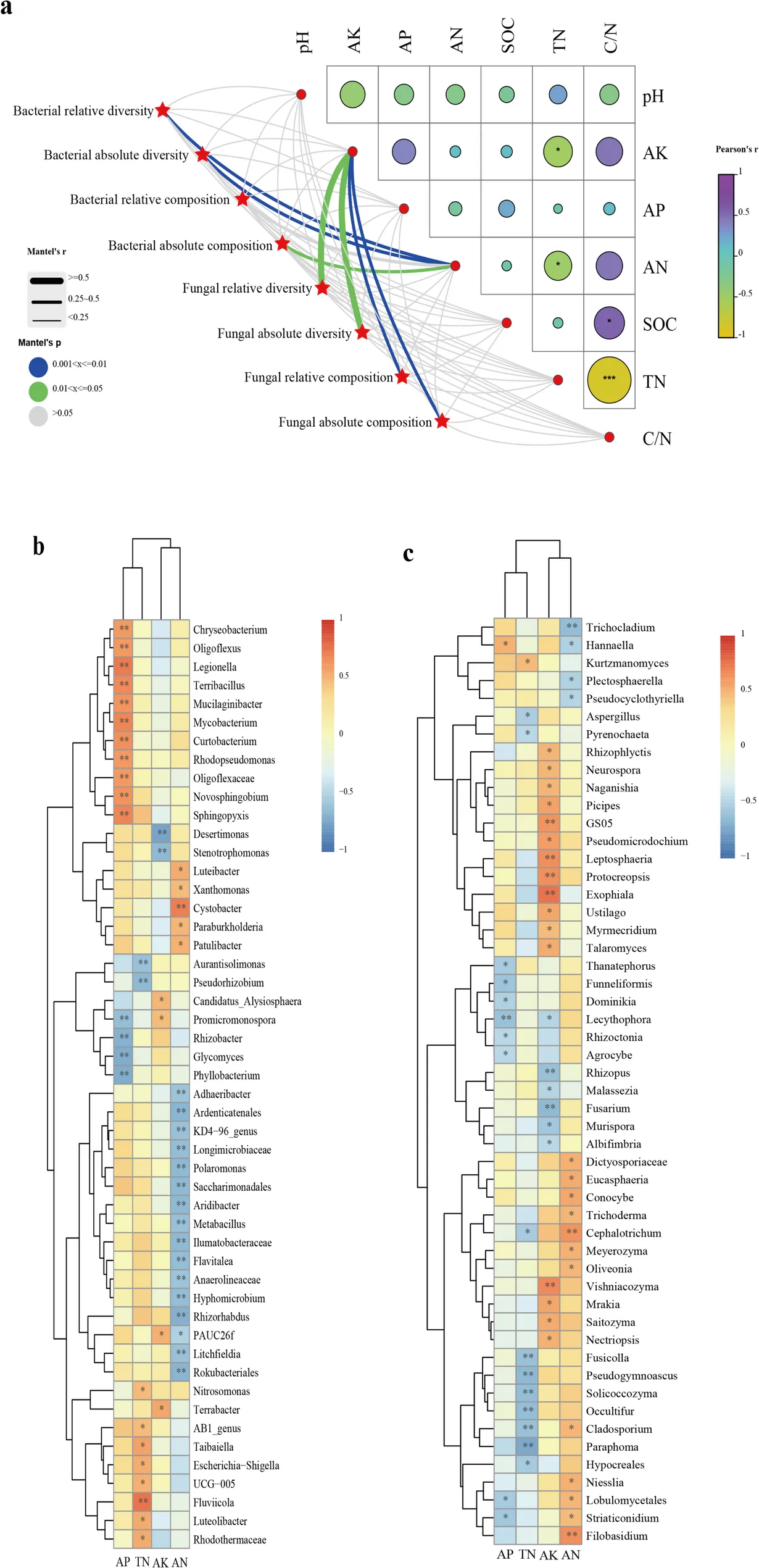
Relationship between bacterial and fungal genera and soil factors based on absolute quantification. Note (**a**): Mantel correlations between environmental factors and bacterial/fungal alpha diversity and community composition. Gray lines indicate no significant Mantel correlation. Green and blue lines indicate correlations significant at 0.01 < *P* ≤ 0.05 and 0.001 < *P* ≤ 0.01, respectively. Figures b and c show heatmaps of the correlations between bacterial genera (**b**) or fungal genera (**c**) and soil nutrient factors (AP, TN, AK, AN). AP: available phosphorus; TN: total nitrogen; AK: available potassium; AN: alkali-hydrolyzable nitrogen. All correlation coefficients included in the analysis have absolute values ≥ 0.3. “*” and “**” indicate significant correlations at *P* < 0.05 and *P* < 0.01, respectively

### Interaction between stover return amount and nitrogen application rate

Two-way ANOVA showed that the effects of stover return (S) and nitrogen application (N) on root endophytic diversity differed clearly between bacteria and fungi. Bacterial diversity indices responded sensitively to both factors, and the interaction (S×N) was generally stronger than the main effects. Fungal diversity indices showed no significant responses.For bacterial communities, the Shannon index (both relative and absolute) was significantly affected by S and N, and the S×N interaction was highly significant (*P* < 0.001). Simpson index and ASV richness were mainly driven by the interaction. Bacterial Observed richness (BR Observed) also showed a highly significant interaction only (*P* < 0.001), suggesting that single measures were not enough to change species richness; their combined effect was needed.In contrast, for fungi, whether based on relative (FR) or absolute (FA) abundance, Shannon index, Simpson index, and ASV richness showed no significant response to S, N, or S×N (*P* > 0.05). Fungal community diversity remained stable under these experimental conditions (Table 1). 

**Table 1:** Interaction of stover return amount and nitrogen application rate on root endophytic microorganisms

Treatment	DF	Straw stover return (S)	Nitrogen level (N)	S*N
		2	1	2
BR Observed	Relativity	NS	NS	***
	F value	2.92	4.57	14.92
	P value	0.09	0.05	0.0006
BRShannon	Relativity	**	**	***
	F value	11.35	9.36	25.39
	P value	0.002	0.01	0.00005
BA Shannon	Relativity	***	*	***
	F value	13.13	7.27	30.22
	P value	0.00095	0.02	0.00002
BA Simpson	Relativity	*	NS	**
	F value	5.32	1.21	12.02
	P value	0.02	0.29	0.00136
BA ASV	Relativity	NS	*	*
	F value	2.74	7.26	5.80
	P value	0.10	0.02	0.02
FR Shannon	Relativity	NS	NS	NS
	F value	0.11	0.06	3.10
	P value	0.89	0.81	0.08
FR Simpson	Relativity	NS	NS	NS
	F value	0.17	0.04	2.41
	P value	0.84	0.84	0.13
FA Shannon	Relativity	NS	NS	NS
	F value	0.03	0.07	1.96
	P value	0.97	0.79	0.18
FA Simpson	Relativity	NS	NS	NS
	F value	0.23	0.06	1.90
	P value	0.80	0.81	0.19
FA ASV	Relativity	NS	NS	NS
	F value	1.69	1.24	1.01
	P value	0.23	0.29	0.39

*BR *Bacteria relatively, *BA *Bacteria absolutely, *FR *Fungal relatively, *FA *Fungal absolutely

“*”,“**”and ‘***” indicate significant correlations at *P* < 0.05,*P* < 0.01 and *P* < 0.001, respectively

## Materials and methods

### Site description and natural conditions

The field experiment was conducted at the Agricultural and Animal Husbandry Science and Technology Demonstration Park of Inner Mongolia Minzu University (Fengtian Town, Horqin District, Tongliao, Inner Mongolia Autonomous Region, China), located at 43°36′ N, 122°25′ E, at an elevation of 182 m above sea level. This area is part of the irrigated region of the West Liaohe Plain. It has a temperate continental monsoon climate. The annual average temperature is about 6.8 °C. The annual growing degree days (GDD) above 10 °C is about 3200 °C. The frost-free period is about 149 days, and the average annual sunshine is about 3000 h. The average annual rainfall is about 390 mm.

The soil type is grey meadow soil. Before the experiment, the topsoil (0–20 cm) had an organic matter content of 17.52 g kg⁻¹, alkali-hydrolyzable nitrogen of 45.20 mg kg⁻¹, available phosphorus of 28.32 mg kg⁻¹, and available potassium of 132.21 mg kg⁻¹.

### Experimental design and field management

A two-factor split-plot design was used. The main plot factor was maize stover return amount, with three levels: no return, half return, and full return. The full and half return amounts were 9000 and 4500 kg·ha⁻¹, respectively. Stover was chopped into 3–5 cm pieces after harvest in autumn and mixed into the soil by deep plowing. The sub-plot factor was nitrogen application rate, with two levels: conventional and reduced. There were six treatments in total: NN (no return + conventional N), NR (no return + reduced N), HN (half return + conventional N), HR (half return + reduced N), TN (full return + conventional N), and TR (full return + reduced N). This was a five-year long-term experiment. Each treatment had three replicates. Plot size was 90 m². Total area was 1620 m².

The maize cultivar used in this study was “Dika 159” (seeds provided by China Seed International Seed Co., Ltd., Zhangye, Gansu; in accordance with GB4404.1‑2006). It was planted using a wide-narrow row (80 cm + 40 cm) pattern with shallow buried drip irrigation. Planting density was 7.5 × 10⁴ plants ha⁻¹. All treatments received the same base fertilizers: diammonium phosphate (18–46–0) at 270 kg ha⁻¹ and potassium sulfate (0–0–50) at 90 kg ha⁻¹. A total of seven irrigations were applied during the maize growing season. The total irrigation amount was 2400 m³/hm². The timing and amounts were: sowing (550 m³/hm²), jointing (425 m³/hm²), large bell (385 m³/hm²), tasseling (385 m³/hm²), silking (275 m³/hm²), filling (190 m³/hm²), and milk ripening (190 m³/hm²). The nitrogen source for topdressing was urea (46–0–0). The total urea amounts for conventional and reduced nitrogen treatments were 450 and 300 kg·ha⁻¹, respectively. Urea was applied in three splits: 30% at V6, 60% at V12, and 10% at R1. Other field management followed local standard practices.

### Sampling, processing, and soil analysis

At the R1 stage of maize in 2025, root-zone soil (0–20 cm) and roots were collected using a five-point sampling method. Three biological replicates were taken per treatment (each replicate was a mixed sample from five points). Rhizosphere soil was air-dried and analyzed following Bao (2000) [29]. Measured variables included available phosphorus (molybdenum blue colorimetry), available potassium (flame photometry), total nitrogen (Kjeldahl method), alkali-hydrolyzable nitrogen (alkaline diffusion method), pH (soil: water ratio 5:1), organic carbon (potassium dichromate oxidation), and carbon-to-nitrogen ratio. Roots were washed with tap water and cut into segments. They were surface-sterilized using 75% ethanol and 2%–3% NaOCl. The final rinse solution was plated to check sterilization effectiveness. After sterilization, samples were stored at − 80 °C.

### DNA extraction and PCR

Total root DNA was extracted using the Fast DNA^®^ Spin Kit for Soil (MP Biomedicals, USA) [30]. The extraction followed the manufacturer’s instructions. Exactly 0.5 g of surface-sterilized root tissue was weighed. This mass was recorded for later calculation of target gene copies per sample. DNA integrity was checked by 1% agarose gel electrophoresis [31]. DNA concentration and purity were measured using a NanoDrop 2000 and Qubit 3.0 fluorometer (Thermo Fisher Scientific, USA). The A260/A280 ratio was between 1.8 and 2.0, and the A260/A230 ratio was greater than 2.0. DNA concentration was then adjusted to 10 ng·µL⁻¹, with a total amount above 500 ng.

Synthetic spike-in internal standards were added to the DNA samples. These standards shared the same conserved regions as natural 16 S rRNA genes but contained identifiable artificial sequences. Nine internal standards were mixed at known gradient copy numbers. They were distributed across four gradient levels (2, 2, 2, and 3 standards per level). The theoretical copy numbers for each gradient were 10³, 10⁴, 10⁵, and 10⁶ copies·µL⁻¹. These were used to construct standard curves.

The V4-V5 region of the bacterial 16 S rRNA gene was amplified using primers 515 F (5′-GTGCCAGCMGCCGCGG-3′) and 907R (5′-CCGTCAATTCMTTTRAGTTT-3′). These primers effectively avoid co-amplification of host chloroplast and mitochondrial DNA. The fungal ITS2 region was amplified using universal primers ITS3 (5′-GCATCGATGAAGAACGCAGC-3′) and ITS4 (5′-TCCTCCGCTTATTGATATGC-3′). PCR reactions were performed in 10 µL volumes containing: 0.1 µL Top Taq DNA polymerase, 1 µL 10× buffer, 0.8 µL dNTPs (2.5 mmol·L⁻¹), 0.2 µL each forward and reverse primer (10 µmol·L⁻¹), 1 µL template DNA (normalized to 10 ng·µL⁻¹), 1 µL internal standard DNA, and ddH₂O to 10 µL. The thermal cycling protocol was: initial denaturation at 94 °C for 2 min; then 17–25 cycles of denaturation at 94 °C for 20 s, annealing at 55 °C for 30 s, and extension at 72 °C for 1 min (25 cycles for bacteria, 22 cycles for fungi); and a final extension at 72 °C for 10 min. Products were held at 4 °C.

The amplified products contained both the target regions from the sample genomes and the internal standard sequences. After amplification, library construction was performed by introducing sample-specific barcodes using high-fidelity PCR with indexed primers. Libraries were purified, quantified, and sequenced on an Illumina NovaSeq 6000 platform with 2 × 250 bp paired-end reads.

### Bioinformatics and statistical analysis

#### Sequence processing, ASV construction, absolute quantification, and taxonomic assignment

Raw paired-end sequencing data were demultiplexed using the Illumina platform. They were then processed using QIIME2 (version 2023.2). Residual adapters and primers were removed with the cutadapt plugin. Quality filtering, denoising, paired-end merging, and chimera removal were performed using the DADA2 plugin. This produced an amplicon sequence variant (ASV) feature table and representative sequences. Taxonomic classification was performed using the classify-sklearn naive Bayes classifier in QIIME2. Bacterial 16 S rRNA sequences were aligned to the SILVA database (v 138.2) with a confidence threshold of 0.7. Fungal ITS sequences were aligned to the UNITE database (v 10.0) with a confidence threshold of 0.6. Taxa below the threshold were labeled “unclassified”.

Absolute quantification used the spike-in method. Nine synthetic internal standards with known copy numbers were added to each sample DNA in a distribution pattern of “2, 2, 2, 3” across four concentration gradients (theoretical copy numbers: 10³, 10⁴, 10⁵, 10⁶ copies·µL⁻¹). Sample DNA and internal standard DNA were co-amplified in the 10 µL PCR system. The amplified products contained both the target regions (16 S rRNA or ITS) and the internal standards. After sequencing, reads from the internal standard ASVs were extracted. A standard curve was built for each sample using the log10 of the known copy numbers. A linear fit with R² ≥ 0.98 was required. The read counts of each sample ASV were then interpolated using the corresponding standard curve to obtain the absolute copy number of that ASV.

The conversion formula for copies per unit of DNA was: absolute copies per ng DNA = (ASV absolute copies) / (amount of template DNA used in sequencing). Then, using the total DNA extracted from the sample and the sample mass used for DNA extraction, the copies per gram of sample were calculated as: absolute copies per g sample = (ASV absolute copies × total extracted DNA amount) / (template DNA amount × sample mass used for extraction).

To correct for the effect of multiple copies of the bacterial 16 S rRNA gene, the rdp-classifier (v 2.14) was used to annotate each ASV at the genus level and estimate its copy number. The absolute ASV copy number was then divided by this estimated copy number to obtain the corrected absolute abundance. For the fungal ITS region, due to large intra- and inter-species copy number variation and the lack of a reliable universal correction standard, no copy number correction was applied.

A total of 3 791 965 raw paired-end sequences were obtained for bacterial 16 S rRNA. After quality control and chimera removal, 3 255 654 valid sequences remained. For bacterial relative quantification, 6 551 ASVs were identified. For bacterial absolute quantification, 6 719 ASVs were identified. The average sequencing depth per sample was 180 870 ± 27 843 reads. For fungal ITS2, a total of 11 743 378 raw sequences were obtained. After quality control, 9 426 030 valid sequences remained. For fungal relative quantification, 1 744 ASVs were identified. For fungal absolute quantification, 1 464 ASVs were identified. The average sequencing depth per sample was 174 556 ± 26 032 reads.

Raw sequencing data have been submitted to the China National Center for Bioinformation. The bacterial data accession number is CRA042941. The fungal data accession number is CRA043519.

#### Community diversity analysis

Based on corrected bacterial (copy number corrected) and uncorrected fungal absolute abundance data, as well as relative abundance data, alpha diversity indices (Observed, Shannon, Simpson) were calculated in R (v 4.2.0). Rarefaction curves were used to assess whether sequencing depth was sufficient. Beta diversity analysis was performed using principal coordinate analysis (PCoA) based on Bray-Curtis distance (for fungi) and Weighted UniFrac distance (for bacteria). Permutational multivariate analysis of variance (PERMANOVA, 999 permutations) was used to test the significance of between-group differences in community structure. PERMDISP tests were also performed to verify whether observed differences were driven by within-group dispersion. These analyses were mainly carried out using the vegan package.

#### Differential taxon and functional prediction analysis

LEfSe analysis (LDA score threshold > 2.0, *P* < 0.05) was used to identify bacterial and fungal taxa that differed significantly among treatments. To validate the LEfSe results, DESeq2 (based on a negative binomial distribution, with P values adjusted using the Benjamini-Hochberg method, α = 0.05) was also used to test for differential abundance at the genus level. Taxa identified by both methods were considered robust biomarkers.

Bacterial functions were predicted using PICRUSt2 (v 2.3.0). ASV sequences and abundance tables were used to predict the abundances of KEGG Orthologs (KOs) and Enzyme Commission (EC) numbers. These were then mapped to the FAPROTAX (biogeochemical cycling) and BugBase (phenotype) databases. Fungal functions were annotated using the FUN Guild and Fungal Traits databases. Differences in relative and absolute abundances of functional groups among treatments were compared using one-way ANOVA and Kruskal-Wallis tests.

#### Environmental factors and statistical analysis

Available phosphorus (AP), available potassium (AK), total nitrogen (TN), alkali-hydrolyzable nitrogen (AN), pH, and soil organic carbon (SOC) were used as environmental factors. A detrended correspondence analysis (DCA) was first performed to select the appropriate model. Redundancy analysis (RDA) was then chosen, based on Hellinger-transformed absolute abundance data at the genus level. Forward selection and Monte Carlo permutation tests (999 permutations) were used to identify environmental factors that significantly influenced community structure (*P* < 0.05). The RDA results show the explained variation for each constrained axis and the contribution of significant factors. Mantel tests (999 permutations) were used to assess the correlation of each environmental factor with community composition (Bray-Curtis distance) and diversity indices (Euclidean distance). For all correlation analyses, P values were adjusted for multiple comparisons using the Benjamini-Hochberg method.

For comparisons of alpha diversity indices and soil nutrient contents among treatments, Shapiro-Wilk normality tests and Levene’s tests for homogeneity of variance were first performed. For variables meeting normality and homoscedasticity, one-way ANOVA followed by Tukey’s HSD post-hoc test was used. For heteroscedastic data, Welch’s ANOVA or Kruskal-Wallis tests were used, followed by Dunn’s post-hoc test. For specific two-group comparisons, the Wilcoxon rank-sum test was used. The significance levels were defined as *P* < 0.05, *P < 0.01, and **P < 0.001. Using stover return amount and nitrogen application rate as fixed factors, a split-plot two-way ANOVA was performed to test for main effects and interactions. F values, P values, and effect sizes (partial eta squared) were calculated. All statistical analyses and visualizations were mainly performed in R (v 4.2.0) using packages such as ggplot 2, pheatmap, and vegan.

## Discussion

### Differential responses of root endophytic bacterial and fungal communities under carbon–nitrogen interaction

This study shows that stover carbon input and nitrogen level change differentially affect the assembly of root endophytic bacteria and fungi. For the bacterial community, under full stover return coupled with reduced nitrogen (TR), sufficient exogenous carbon and relatively low available nitrogen may increase resource heterogeneity, allowing more functional groups to coexist. This weakened the absolute dominance of eutrophic *Pseudomonas* and promoted colonization by taxa with associative nitrogen-fixing capacity (*Rhizobium*, *Agrobacterium*) and complex organic matter-degrading ability (Actinomycetota). This result supports the theory that resource heterogeneity promotes niche differentiation [32–33]. In contrast, under no stover return with reduced nitrogen (NR), the extreme scarcity of degradable carbon sources enabled *Pseudomonas*, capable of rapidly utilizing inorganic nitrogen, to outcompete other species, leading to significantly reduced community diversity and structural simplification [34]. Half stover return with reduced nitrogen (HR) exhibited a trend toward functional specialization, significantly enriching Bacteroidota involved in cellulose and hemicellulose degradation [35–36], suggesting that moderate carbon input may generate a directional screening pressure for specific functional groups through the accumulation of particular degradation intermediates [37].

For the fungal community, nitrogen reduction was a key driver of compositional changes, and the addition of stover return further amplified this differentiation effect. Unlike the dramatic fluctuations in bacteria, fungal alpha diversity remained relatively stable across all treatments. This may be because fungal hyphal networks and strong saprotrophic capacity allow them to maintain diversity through species turnover (e.g., shifts in abundance of *Fusarium*, *Actinomucor*) under nitrogen stress, rather than directly exhibiting reduced diversity indices [38–39]. In treatments without stover return, the proportion of potential pathogens such as *Fusarium* increased, suggesting that carbon deficiency may drive some fungi to utilize living root exudates, thereby increasing disease risk [40–41]. In contrast, TR and HR strengthened the organic matter transformation function of the fungal community by enriching saprotrophs such as *Mortierella* and *Penicillium* [42], further highlighting the role of coupled carbon and nitrogen management in shaping the functional orientation of the fungal community.

### Trade-offs among microbial diversity, functional potential, and community assembly

Under coupled carbon and nitrogen management, root endophytic microbial communities of maize exhibit complex trade-offs among diversity maintenance, functional enhancement, and community stability. In TR, full stover carbon input not only alleviated the inhibitory effect of nitrogen reduction on bacterial Shannon diversity but also synergistically enhanced multifunctional potential, including nitrogen fixation and organic matter transformation. This finding is consistent with the theoretical expectation of a positive relationship between biodiversity and ecosystem multifunctionality [43–44]. In this treatment, ample exogenous carbon likely buffered the negative impact of nitrogen limitation on community evenness by expanding resource dimensionality and niche space [45], thereby achieving simultaneous improvements in both diversity and multifunctionality. HR maintained relatively high diversity while enhancing specific functions such as hydrocarbon degradation, indicating a coexistence of functional redundancy and functional specialization [46]. However, this long-term directional selection toward specific degradation functions may potentially weaken community functional redundancy and system resilience, posing a risk of reduced ecological functional stability [47–49].

The functional response of the fungal community was primarily reflected in the restructuring of trophic modes. In HR and TR, the abundance of Basidiomycota, a phylum associated with lignin degradation, increased, consistent with the functional role of this group in the transformation of recalcitrant organic matter [50–51]. This suggests that stover return under nitrogen reduction reinforces the functional module of fungal involvement in recalcitrant substrate transformation. In contrast, in treatments without stover return, such as NN and NR, the proportion of pathogenic fungi (e.g.,*Fusarium*) increased, indicating that in the absence of stover carbon input, the fungal community may shift toward utilizing root exudates or living tissues, thereby increasing the potential risk of soil-borne diseases [52]. These results reveal that the maize root endophytic microbial community is a highly differentiated and dynamically balanced system, and that carbon and nitrogen management must be precisely regulated to achieve an optimal balance between functional enhancement and diversity maintenance.

### Alkali-hydrolyzable nitrogen and available potassium drive community assembly

In this study, redundancy analysis and Mantel tests consistently indicated that alkali-hydrolyzable nitrogen (AN) and available potassium (AK) were the core environmental factors driving the divergence of maize root endophytic bacterial and fungal communities. Moreover, the regulatory weights of these two factors differed significantly between bacteria and fungi, revealing niche differentiation in resource acquisition strategies among root endophytes. As an instantaneous indicator of soil nitrogen supply intensity, AN imposed significant deterministic selection pressure on the bacterial community [53]. By altering the nutrient flux within roots, this selection pressure drove directional turnover between eutrophic and oligotrophic taxa, thereby forming a co-occurrence network oriented by nitrogen availability [54]. In contrast, the fungal community showed a much weaker response to AN and was more strongly filtered by mineral elements such as AK, reflecting fundamental differences in the resource acquisition strategies of root endophytes [55–56]. Potassium is the most abundant cation in plants and is involved in osmotic regulation, enzyme activation, and signal transduction [57]. Changes in potassium availability can also indirectly impose selective filtration on root-colonizing microorganisms by altering the composition and secretion rate of root exudates. In this study, functional taxa such as *Promicromonospora*, *Terrabacter* (bacteria) and *Neurospora*, *Talaromyces* (fungi) were significantly positively correlated with AK, suggesting that available potassium may act as an “ecological filter“ [58–61], directionally enriching beneficial microbial taxa. Conversely, potential pathogens such as *Fusarium* and *Rhizopus* were significantly negatively correlated with AK, further confirming that insufficient potassium supply may deteriorate root microecological health [62].

Stover return has been shown to promote the release of mineral potassium from soil and the rapid cycling of potassium from stover [63], thereby effectively increasing soil available potassium content [64]. In this study, under long-term stover return treatments (TR and HR), changes in soil AK level were significantly correlated with shifts in bacterial and fungal community composition, indicating that the synergistic effect of stover return and nitrogen reduction was partially achieved through the optimization of potassium supply [65]. This finding reveals an indirect regulatory pathway of carbon and nitrogen management on the root microbiome, whereby altering potassium cycling status triggers a nutrient cascade effect that reshapes the assembly pattern of the root endophytic community. This underscores the critical role of potassium in maintaining microbial diversity and functional stability. Given the widespread occurrence of potassium deficiency or potential potassium deficiency in northern Chinese farmland, integrated nutrient management strategies should give adequate attention to potassium supply, not only for crop yield and quality but also for the health and functional stability of the root microecosystem.

### Research significance and limitations

This study systematically revealed the combined effects of stover return and reduced nitrogen management on the community structure, diversity, and potential functions of maize root endophytic bacteria, providing new insights into plant–microbe interactions under agricultural management practices. However, as this study was based on a single sampling period, the generalizability of the findings and their actual contribution to crop yield require further validation through multi-year, multi-growth-stage dynamic monitoring.

## Conclusions

Under the conditions of this experiment, different combinations of stover return and nitrogen application exerted differential effects on the assembly of maize root endophytic microbial communities. The treatment combining full stover return with reduced nitrogen application (TR) decreased the relative abundance of the dominant bacterial genus *Pseudomonas*, while significantly enriching *Rhizobium* and *Agrobacterium* (with associative nitrogen-fixing capacity) and Actinomycetota (with complex organic matter degradation potential), and promoting the colonization of saprotrophic fungi such as Mortierella; Suggests that coordinated carbon and nitrogen input may be conducive to the formation of a multifunctional microbial community. In contrast, the half stover return with reduced nitrogen treatment (HR) specifically enriched Bacteroidota, yet bacterial diversity showed a declining trend. Meanwhile, in the no stover return with reduced nitrogen treatment (NR), *Pseudomonas* became absolutely dominant, bacterial community structure became homogenized, and while absolute fungal abundance increased, it was characterized by a high concentration of a few potentially pathogenic genera such as *Fusarium*, indicating an elevated ecological health risk.

Two-factor analysis showed a significant interaction between stover return and nitrogen application rate on bacterial diversity, whereas fungal diversity remained relatively stable under the same treatments, suggesting divergent response strategies to environmental changes between the two microbial groups.Soil alkali-hydrolyzable nitrogen and available potassium were identified as environmental factors closely associated with root endophytic microbial community divergence: alkali-hydrolyzable nitrogen showed a strong correlation with directional turnover of the bacterial community, whereas available potassium was linked to the enrichment of multiple beneficial genera and a lower abundance of potential pathogenic genera such as *Fusarium*.In conclusion, full stover return combined with reduced nitrogen application (TR) may promote the assembly of root endophytic microorganisms with both nitrogen-fixing and organic matter transformation potential through optimizing carbon, nitrogen, and potassium resources, representing a promising agronomic strategy for sustainable maize production in the West Liaohe Plain irrigation area.

## Data Availability

The datasets used and/or analyzed during the current study available from the corresponding author on reasonable request.
